# Inversio-Uteri—Its Causes—Mechanism and Medico-Legal Bearings

**Published:** 1861-01

**Authors:** N. S. Davis

**Affiliations:** Prof. of Principles and Practice of Medicine in the Medical Department of Lind University, and Prof. of Clinical Medicine in the Mercy Hospital, Chicago, Ill.


### INVERSIO-UTERI—ITS CAUSES—MECHANISM AND MEDICO-LEGAL BEARINGS.

By N. S DAVIS, M. D.,

Prof. of Principles and Practice of Medicine in the Medical Department of Lind University, and Prof. of Clinical Medicine in the Mercy Hospital, Chicago, Ill.

Having been recently compelled to occupy the witness stand in the Circuit Court of this city, for more than two days, under examination on the subject of Inversion of the Uterus, I have thought it might not be unprofitable to the readers of the *Examiner*, to publish the testimony there given, more especially as the opinions therein expressed are the result of a previous careful investigation of the subject in all its relations. I am enabled to do this with facility from the verbatim notes of a professional reporter.

Inversion of the Uterus, is not only one of the most serious displacements or causalties to which the lying-in woman is subject; but in the present state of medical literature, it can hardly occur in the practice of any physician, without subjecting him to serious suspicions of mal-practice; and if the case should unfortunately become the subject of legal investigation, it will almost certainly result in confirming such suspicions, and consequently blasting a reputation which had cost its possesser twenty or thirty years of hard professional toil to establish. This is owing chiefly to three circumstances, that will become apparent to every one, who candidly enters upon an investigation of the subject.

1st. The former very general custom of employing Midwives in obstetrical practice, instead of educated physicians, and the natural tendency of the latter when called to a case of inversion already existing, to attribute it at once to the ignorance and injudicious pulling of the attending Midwife. Thus of sixty-four cases of *inversion* to be found within my reach, in which the causes are stated by those reporting the cases, thirty-eight were attended by Midwives, and of this number twenty-nine are said to have been produced by *traction* on the cord. While of the twenty-six attended by physicians, only eight are alleged to have been produced by traction on the cord or forcible efforts to deliver the placenta. So great a desparity in the relative proportion of cases, occurring from the same alleged cause can scarcely be regarded as accidental.

On the contrary, it affords strong presumptive evidence, either that the Midwives have been, in many instances at least, accused of forcible traction and inversion without any other proof than the fact that an inversion was subsequently found to exist; or that physicians have been unfaithful in reporting their own cases, by purposely concealing the true cause.

The latter supposition necessarily impeaches the integrity of those members of the profession who have reported cases of inversion occurring in their own practice, and hence cannot be entertained for a moment by any candid man.

For we not only find among them men of the highest eminence and of unimpeachable varacity, but their reports show beyond all cavil, that inversions do take place without traction on the cord or any other kind of interference whatever. Thus we find several cases in which the inversion followed during the same pain that expelled the child, and consequently before any interference could have taken place by the attendants.

In several other cases the inversion occurred during the first pain succeeding the expulsion of the child, and while the attendant was neither touching the cord nor any part of the person of the mother. Such was the case reported by Prof. D. H. Storer, in the Boston Medical and Surgical Journal several years since. But if it is conceded that cases have occurred thus spontaneous, or without the slightest interference on the part of the attendants, and in the presence of such men as Denman, Radford, Storer, &c., there is no possible reason why other cases may not occur in the same way in the presence of the most obscure practitioner in the country, or even in the hands of the most ignorant Midwife.

It is very apparent, therefore, that the mere presence of an inversion is not fair presumptive evidence that violence has been done, by pulling on the cord or in any other way, and as a large proportion of the cases attributed to the action of Midwives, rests on no other foundation, they should be rejected from all attempts to estimate the importance of traction on the cord as a cause of inversion. If we follow this rule, and take only those cases which have been reported on the authority of the physician or midwife actually in attendance, we shall find the number attributed to traction on the cord or any other undue measures for removing the afterbirth, proportionately small; indeed scarcely more than twenty-five per cent of the whole number. This we admit, is a widely different result from that stated by Dr. C. A. Lee, in a statistical article on Inversio-Uteri, in the *American Journal of Medical Sciences*, for October 1860. On page 344 of that article, Dr. Lee says: "Accordingly, we find that of the 148 cases, of which an abstract is above given, the cause is assigned in only 62 cases, and of those 39 are stated *to have been attended by midwives*, a large majority of them in Europe. In 39 cases, moreover, we are expressly informed that the inversion occurred *from pulling on the cord.*" He then specifies by enumeration these 39 cases; but on referring to the cases thus specified we find no less than 10 of them destitute of any such allegation as "*pulling on the cord.*" One of these 10 cases, is the noted one reported by Dr. Skae, in which the inversion occurred after an abortion taking place at the fourth month of utero-gestation. Another is the case reported by Dr. Storer, in which he says: "in five minutes after the child was born, the placenta was expelled, the cord *not being* touched except to divide it. On examination, the uterus was found inverted with the placenta attached." In making up important statistics an error of 10 in 39, is quite a distance from mathematical accuracy. Again, on page 358 of the same article, Dr. Lee says: "We have seen above that of the *fifty-two* cases in which the cause of inversion is assigned, *thirty-eight* are distinctly stated to have occurred from traction on the cord, &c." These are but a small share of the gross errors and discrepancies contained in this article of Dr. Lee; but enough surely to render his deductions entirely unreliable. Passing by these discrepancies, however, and examining in detail the 38 or 39 cases alleged by Dr. Lee, to have occurred from pulling on the cord, we find 29 of them, to have been attended by *midwives*; and not one of which was reported directly on the authority of the midwife herself. And yet she was the only one who could actually know whether any traction was made on the cord or not.

2d. The second circumstance that presents a very strong barrier against the physician in a court of justice, consists in the *assumption* on the part of many eminent writers, that the simple existence of an inversion is prima facia evidence of mal-practice on the part of the attending physician or midwife. Thus Dr. Robert Lee, of London, says: "Inversion of the uterus is frequently, if *not invariably*, the consequence of pulling on the cord, to extract the placenta, &c." Colombat says: "The most common course of inversion consists in attempts to deliver the placenta immediately after the birth of the child," &c. Ramsbotham says: "Whenever this serious accident has happened, it may *generally* be looked upon as the consequence of *improper* treatment." And Dr. Chas. A. Lee, in the article to which we have already alluded, says: "Where an inversion is first detected some days, weeks, months, or years after delivery, blame is very apt to attach to the attending physician, and the censure, implied or expressed, is with very few exceptions, *undoubtedly deserved.*" It is true that the truth of these statements, is either doubted or denied by equally high authority, such as Tyler Smith, Radford, Simpson, Meigs, &c., yet it does not prevent their exerting a strong influence. And still a little reflection will satisfy any candid investigator, that no statement could be more unphilosophical, or less supported by well ascertained facts. They are unphilosophical because they make the simple existence of an inversion, proof that undue violence has been used in the removal of the placenta, and therely *beg* the whole question at issue. Having done this, they write down every case as occurring from traction on the cord or violence in the removal of the placenta, that happens to occur in the hands of a midwife or an obscure practitioner, and then adduce these same cases as numerical proof of the correctness of their first assumption. Could any pretended series of cases be less reliable, or any process of reasoning more absurd? But Dr. Chas. A. Lee, in his recent paper on this subject, goes still farther, and on page

344 uses the following language, viz: "In analyzing the above cases, it is to be regretted that many of the original reports are so imperfect, that we are often left to *surmise* the cause of the accident. This is what might, perhaps be expected; for, if it is an accident—as many writers allege—which may, with proper care and skill, *always be prevented*, then it is not to be presumed that the practitioner who places much value on his reputation, will be forward to acknowledge that so serious an accident has resulted from his ignorance or neglect. Hence, also, we may look, not unfrequently, for cases of *spontaneous inversion*—cases in which the womb, without cause, provocation, or premonition, turned itself inside out, all at once, some days, weeks, or months after delivery, on going to stool, straining, laughing, crying, singing, walking, or other kinds of exertion, or no exertion at all. And we are asked in all seriousness, to wonder at such strange anomolies, and ask why nature should enact such freaks without special object."

Here we have, first, a direct intimation that inversions, can "always be prevented," by proper care and skill on the part of the attending physician; and second, that those in whose care inversions have occurred, have been guilty of making false reports, by omitting to state the true cause and alleging spontaniety of occurrence, for the purpose of shielding their own reputations. These charges are certainly more grave than modest; and it may not be amiss to ascertain to whom they apply. In simply looking over the list of cases contained in Dr. Lee's own article, we find one case of inversion occurred in the practice of Dr. Robert Smith, two in that of Dr. A. Fisher, one in that of Dr. Mackay, of Buffalo, one in that of Dr. Geo. J. Fisher of Sing Sing, one in that of Dr. J. G. Forbes, one in that of Dr. E. Fisher, one in that of Dr. W. L. Sutton, one in that of Dr. D. H. Storer, of Boston, one in that of Dr. W. J. Square, one in that of Dr. Lever, two in that of Dr. Radford, one in that of Dr. Denman, one in that of Dr. P. Bissel, one in that of Dr. Dewees, one in that of Dr. Teallier, &c. According to the modest intimation of Dr. Charles A. Lee, all these and many others must have been deficient in "*care and skill*," and then guilty of making dishonest reports to shield their reputations.

Could a man place on the pages of our medical literature, statements or intimations more wantonly unjust, or better calculated to defeat the ends of justice? In nearly all the cases of inversion occurring at the time of delivery, the placenta has been found adherent; and in several of the cases, the inversion occurred with the same pain that expelled the child. Will Dr. Lee, or any one else inform us how much "care and skill," the physician must have to enable him to know before the child is expelled, whether the placenta will remain adherent or not, or whether the uterus is *about to be expelled with the child?* The facts on record are abundantly sufficient to satisfy every candid mind, that numerous cases of inversion have occurred strictly *spontaneous*, that is, without any interference whatever on the part of the attending physician.

Dr. Lee himself, acknowledges *twenty-three* cases of this kind among those quoted in his article. Hence, instead of assuming that the existence of an inversion necessarily implies want of "care and skill" on the part of the attendant, every just rule of criticism would require us to assume just the reverse, it being a universally acknowledged maxim, both in law and morals, that every man must be presumed innocent until he has been clearly proven guilty.

3d. The third and last circumstance that we shall name as having a tendency to obscure this subject and increase the difficulty of arriving at the truth, either legally or scientifically, is the very general assumption, on the part of writers, that all inversions of the uterus must be commenced if not completed either at the time or within a few hours after delivery. Thus Dr. C. A. Lee, in the article already alluded to says: "*In all cases there can be no doubt whatever, that the first step in the process took place at or near the time of delivery.*"

Of course, the necessary inference from this assumption, is, that in all cases in which the inversion is not detected until some days, weeks, or months have elapsed after the delivery, the attending physician has been guilty of gross carelessness or want of skill, in the management of the case. Neither Dr.

2

Lee, nor any one else denies the fact, that there are many cases of inversion on record, that were not detected or known to have existed until a period of time varying from three days to fifteen or twenty years after the last delivery. And not only so, but in many of them, no symptoms are known to have occurred which afforded the least indication of an inversion, until several days or weeks had elapsed after the delivery. Thus in the case reported by Dr. J. P. White, as occurring in the practice of Dr. Mackay, of Buffalo. So little did the symptoms indicate any trouble soon after labor, that Dr. White says: "This was probably spontaneous; *it certainly was not suspected until three weeks after labor*, and seemed the result of violent bearing down pains, which were promoted, if not induced by imprudent exertions on the part of the patient." Again in the case reported by Dr. W. J. Squire, in the *Provincial Med. and Surg. Journal*, Vol. 1, there was not a symptom indicating the slightest displacement during the first eight days. There was neither hemorrhage, unusual vaginal discharges, nor fullness on bearing down, but on the evening of the eighth day, being quite well, the patient dressed and got up. This was followed by slight flowing, and two days after, while up, the flowing recurred more profusely, and from that date green vaginal discharges with periods of hemorrhage continued with well marked indications of inversion, until the 21st day, when the inversion was detected by a vaginal examination. Commenting on this case, Dr. C. A. Lee, with his accustomed coolness of *assumption* says: "This case, though called spontaneous, is doubtless to be ranked with those where inversion *is began* by traction on the cord."

If the inversion was began by traction on the cord, it must have been by indenting the fundus at the time of delivering the placenta. If the fundus was then indented or depressed so as to commence the inversion, and remained in that condition during the eight days that the patient remained quiet, as supposed by Dr. Lee, it is evident that the uterus must have remained all that time, also in a state of atony or complete quiescence, because, if the usual contraction and involution of the organ had been progressing during that time, the depressed fundus must of necessity have been replaced, or else grasped by the contracting body, and forced on the complete inversion. If the uterus did remain *uncontracted* so as to permit a continuance of the depression, what prevented a continuous hemorrhage? How, with the uterus in a state of atony and the fundus depressed could the patient, not only be free from hemorrhage but also free from any unusual vaginal discharge, and well enough to dress herself and get up ? Again, if it is possible for the uterus to remain after delivery, eight days in so quiescent a condition as not to disturb a depression of the fundus produced by traction on the cord, why would not the same condition permit the pressure of the abdominal viscera, aided by the action of the abdominal muscles, to commence a depression, de novo ? It is plain to the reflecting mind, that the same atonic condition of the uterus that would allow a depression of the fundus to remain in statu quo, from one to three weeks, would also allow a depression to take place de novo, during any part of that time. Hence, we claim that it is contrary to every rule of philosophical inquiry, and equally contrary to every principle of justice to *assume*, without proof, that because an inversion is discovered to exist at some period of time more or less remote from the date of delivery, that it must necessarily have began at the time of such delivery. Besides the cases reported by Dr. J. P. White, and Dr. W. J. Squire, in which the inversion was discovered some time after delivery, others still more striking have been reported by Ana Baudeloeque, Skae, H. Davies, Teallier, and Fisher. Viewing the subject of inversio-uteri in its medico-legal aspect, the very important question arises : shall we with Dr. Lee and others, *assume* that the existence of an inversion is *prima facia* evidence of mal-practice—that the majority of those who have reported cases occurring in the practice of respectable members of the profession, have either omitted or misrepresented the facts, for the purpose of shielding the reputation of the practitioner—and that in all cases, inversion must necessarily *begin* at the time, or within a few hours after delivery, whether there is any evidence of such beginning or not ? Or shall we take the facts and cases as they are presented to us on the pages of our Medical Literature, allowing each respectable member of the profession who reports a case, the same credit for candor and integrity that we claim for ourselves, until he is in some way clearly proven to be destitute of those desirable qualities.

We think there can be but one answer to these questions among right minded men. The latter question clearly indicates our duty, whether as a witness upon the stand, a statistician, or a simple scientific investigator.

Acting on this rule, we have patiently examined all the reported cases of inversion within our reach, with a view to determine *when* and *how* they occurred. We find them capable of being arranged into three classes.

1st. Those that are known to have occurred at the time of the delivery of the placenta.

2d. Those that occurred after the delivery of the placenta, and before the usual time for the muscular contraction of the uterus to cease; that is, within four days after delivery.

3d. Those that were not known to exist until after the ninth day from the delivery.

It will be observed that we include no cases supposed to have occurred between the fourth and ninth days after delivery; simply because we have found none such upon record. This is a fact worthy of careful attention, as it relates to a period of time after the uterus, in ordinary cases has ceased to diminish by active muscular contraction, and during which the process of involution is actively progressing. In the first class of cases which embraces about three fourths of all those on record, adhesion of the placenta, either partial or complete, seems to be a constant accompaniment.

The views we entertain in regard to the mechanism of the cases of inversion belonging to the several classes named, are fully explained in the following testimony, given in the case of Dr. A. Fisher *vs.* H. O. Stone,

CIRCUIT COURT—AFTERNOON SESSION.

*Dr. N. S. Davis* recalled by the plaintiff, and further examined by Mr. Roberts as follows:

*Q.* You are still practicing medicine, Doctor, and still occupied with the said University?

*A.* Yes sir, I occupy the Chair of Practical Medicine there, and Clinical Medicine in the Hospital. I have two distinct duties to perform; one is to teach Practical Medicine in the College, and the other, what we call Clinical Medicine at the bedside of the patient in the Hospital.

*Q.* Have you ever seen any other case of inversion of the womb besides this?

*A.* I have seen one other certainly complete, and one partial.

*Q.* How many degrees of inversion of the womb do you make, and what are they?

*A.* I consider there are three degrees of inversion. One of indentation. Indentation is a depression of the fundus of the womb, not sufficient to be grasped by the other parts below. Partial inversion is that in which the process of inversion is carried far enough for the fundus to be within the mouth of the womb, at, or actually engaged in it. The third degree is where the whole body of the womb is without the os or mouth. I include the neck, except that which is folded immediately at the mouth.

*Q.* State under what conditions in your opinion, inversions usually occur?

*A.* I think inversions usually occur under three essential conditions of the womb. The first is that which takes place at or immediately after delivery. By immediately after delivery, I mean that which takes place at the expulsion of the afterbirth. I think that when inversion occurs at that time, it is produced by a combination of forces. As the head and body of the child pass out of the womb, they necessarily cause the mouth to be widely dilated, and at that act, as the child is expelled, it is as widely dilated as the size of the body itself, or more widely. Then its diameter is as great as that of the body of the womb. In the act of expulsion, the final expulsion of the child, which is the result of contraction, all these fibres or parts of the womb which would diminish its longitudinal or vertical length are contracted, while those that are circular, or would contract its diameter below, are relaxed and passive. It is well known there are two distinct modes of action in the womb, that usually at the commencement of labor, or its early stage, both of these acts exist; that there is a contraction of what we call the longitudinal fibres which are discribed by the most accurate anatomists as a broad band extending from the neck over the fundus, and down the pos terior side near to the neck again. One action, is the contraction of this band, together with the oblique and concentric layers of fibres, that more immediately surround and attach to the Fallopian tubes which are at the upper and back part of the womb. Explaining it by a Blackboard is a very familiar mode of doing it. [A Blackboard was here brought into the Court Room, upon which the witness drew two diagrams. The first representing the impregnated uterus immediately prior to parturition. The second representing it with the os fully distended at the moment of delivery.]

*Witness.* I have drawn there two diagrams. This first one is intended to represent the womb in its development before the fœtus is expelled. I do not pretend to exact accuracy in mathematical length and breadth, but to represent the outline of the womb at its full period of development, with the mouth here a little dilated as in the early stage of labor. The other one represents the womb at the time the child is expelled from it. According to the representation, suppose the neck of the womb was here, [Indicated]. When it is completely developed it is lost in the extension of the organ. It only has a neck in the unimpregnated state. These fibres commence at the original neck of the womb, and pass over the fundus down to near the same point on the opposite side, and are spread out so as to reach nearly from the Fallopian tube on the one side to that on the other. There are fibres here, [indicated,] running in various directions; some obliquely down towards the mouth, others obliquely towards the center, and each side. Then there are other circular fibres more interior and interla.

cing in every direction in the more interior layer of the substance of the womb. When labor has commenced, it is usually the case, that all the fibers of the womb contract with each pain periodically. The result is the longitudinal fibres draw down the fundus with these oblique ones; they contract the fundus in every direction, hugging down on the child. This pushes the child, and the waters with it down to the mouth, but the mouth aud the circular fibres are found to contract at the same time, so that each pain will hug the head, or parts that come down there; or if the finger is inserted to find out what is going on, you will find that with each pain it compresses the finger, but as it relaxes this will become softer, and more dilated. As the labor progresses the longitudinal and oblique fibres seem to master the circular ones, and eventually, as the contents and the water are crowded down here, and begin to appear through the opening it dilates little by little with each pain.

After a while the contraction of the circular fibres becomes more feeble, and the mouth widens each way until it admits not only the waters, but the head of the child, to become engaged in it. The circular fibers now loose their contractility for the time being, and allow the mouth of the womb to become entirely relaxed. The longitudinal and oblique fibres, however, continue to act with increasing force, aided by the voluntary efforts of the patients, until the child is expelled from its cavity. In examining the cases of inversion that are on record, I find that in a considerable number, the placenta and uterus have been expelled with the same protracted and severe pain that expelled the child; thus accomplishing the inversion before the attending physician could have the slightest opportunity to interfere with it.* I can best explain the manner in which such an inversion is produced, by recurring to the figure upon the black-board representing the uterus as the child is being expelled from it. The placenta being usually attached to the inner surface of some part of the body or fundus of uterus, and possessing no power of contraction itself, in

* See cases reported by Denman, Robert Smith, Walter Chaning, E. Fisher, and W. L. Sutton.

ordinary cases of labor, the last pain that finishes the expulsion of the child is usually accompanied by so great a contraction of the whole fundus and body that the placenta is detached. Hence, in ordinary cases we find a gush of blood following the child and immediately after, the placenta either partially or wholly detached lying here in the mouth of the womb and sometimes in the vagina. But suppose the placenta attached here to the fundus of the womb, to be unusually adherent, so that the contraction of the longitudinal and oblique muscles of the fundus does not detach it; you easily percieve that the strong contraction of the broad longitudinal layer following down the exit of the child, would of necessity fold the adherent placenta upon itself, dimpling the fundus in with it, while the strong pressure of the abdominal muscles from the straining of the patient at the same moment would be sufficient to carry the depressed fundus rapidly on to complete expulsion. Such is the mode of inversion when it follows immediately the expulsion of the child, and by the same protracted pain.

But the larger number of cases on record are described as occurring during the first pain that succeeds the expulsion of the child, varying from five to thirty minutes after the latter event.* In all these cases the placenta seems not to have been detached with the pain or contraction of the womb that expelled the child. We must hence suppose it remains attached here to the inner surface of the fundus, until the next pain or contraction. To explain these cases, I shall simply suppose that the neck and mouth of the womb remain in the same state of relaxation as when the child was being expelled from them. That while thus relaxed at the mouth, and the placenta adherent to the inside of the fundus, another pain comes consisting of the contraction of the longitudinal broad muscle over the fundus, and the oblique ones connected with the fallopian tubes, with the simultaneous contraction of the abdominal muscles and straining of the patient, precisely as when the child was expelled. By this process, the fundus is drawn down, doubling

---

* See cases in Ashwell on Females, pp. 402, 410 and 411. Also in Churchill, p. 373. Also a case by D. H. Storer, in New England Medical Journal for 1842. Another by P. Bissell in Transactions of New York State Medical Society for 1859, &c., &c.

the adherent placenta upon itself, aided by the full force of the abdominal pressure above, with no resistance from the relaxed neck and mouth below, until the placenta and womb are both expelled together; constituting a complete inversion. By such a combination of circumstances, the inversion, whether partial or complete, may take place either immediately following the expulsion of the child, or at any succeeding pain while the placenta is adherent and the mouth and neck remain relaxed.

It will be seen by the foregoing explanations, that we regard the coincident presence of four things as necessary to the production of inversion during the expulsion or delivery of the placenta, namely: a perfect relaxation of the neck and mouth of the womb; an adherent placenta; an active contraction of the broad longitudinal layer of muscular fibres over the fundus; and an equally active contraction of the abdominal muscles making a strong downward pressure directly upon the fundus of the womb. These four circumstances existing coincidently, appear to me to have produced a large majority of the cases of inversion on record spontaneously, that is, without any interference on the part of the attending physician or midwife. If while these circumstances co-exist, the attendant makes traction on the cord or in any other way exerts a downward force upon the adherent placenta, it will, of course facilitate the inversion. On the other hand, if these circumstances do not co-exist; if, for instance, instead of entire relaxation of the neck and mouth, all parts of the womb contract naturally as the child passes out of it, the circular fibres of the lower part of the body and neck will contract the lateral diameter simultaneously with the contraction of the fundus by the longitudinal fibres—the consequence of which will be that the fundus being supported by the well contarcted neck and lower part of the body, cannot be inverted by any ordinary force of traction applied to the cord. Indeed, the cord may be torn off, as has been done in several instances, without producing any degree of inversion. Hence, we cannot agree with those writers who represent "pulling on the cord," as the most common cause of inversion; first, because there is no positive proof that any such traction was made in more than twenty-five per cent of the whole number of cases on record ; and second, because such traction, when made, could only be effectual as an incidental aid to the other circumstances we have mentioned. The foregoing explanations relate only to the first and most numerous class ef cases of inversion, that in which the accident occurs during the delivery of the placenta or afterbirth.

But I have said that there were two other classes of cases on record, and consequently two other methods by which inversion may be produced. Both of these occur after the expulsion of the placenta. The one class includes all such cases as occur between the expulsion of the placenta and the fourth day afterwards, while the other inclndes those that appear to have taken place at some period after the ninth day from the delivery. So far as I am able to form an opinion from a careful study of the cases belonging to the second class, they result from a direct downward pressure exerted on the fundus of the womb, while the whole organ is in a state of atony or relaxation after the placenta had been expelled.* It is well known to every experienced practitioner, that the womb may contract so as to expel the child and the placenta in the natural manner, and yet, at a subsequent time, varying from half an hour to three days, perhaps, the womb looses its contractility and becomes again relaxed. This will be likely to be attended by a renewal of hemorrhage, unless prevented by a large coagulum of blood in the uterine cavity. In a time varying from a few hours, (I think the shortest time mentioned in any case under this head, is nine hours,) to three days after the delivery of the child, the womb is capable of becoming sufficiently relaxed to admit of an inversion. In that relaxed condition, coughing, sneezing, rising upright in bed, straining at stool, or any act by which an active force is thrown upon the fundus, is capable of indenting it and forming the commencement of an inversion. Every accoucheur who

---

* See case by M. Roussel, in Cazeaux's Midwifrey p. 918. Also a case by J. G. Forbes in Med. Churg. Trans. Vol. 35, p. 127. Also a case by E. P. Bennett in the American Journal of Medical Sciences, for 1857. Also a case in Radford's Essay, &c.

has had his finger in the vagina at the time, knows the force with which a cough or sneeze propels downward by the active pressure given to the contents of the abdomen

If the fundus of the womb, at any time before muscular action naturally ceases, and the process of involution begins, (which is supposed to be about the fourth day after delivery,) is in a state of entire relaxation, any of those acts that the patient might make, such as coughing, sneezing, singing, straining at stool, &c., would throw a weight or force upon the fundus sufficient to depress it. When once it has done this, it may go on *gradually* increasing the depression, as the successive acts of the patient bring renewed pressure upon it, until the fundus reaches the mouth or even protrudes through it. More generally when the fundus becomes much depressed, its contact here, (referring to diagram on the black-board) with the inner surface of the lower part of the body, excites the circular fibres to contract, by which the fundus is grasped, as it were, and forced on in the downward direction, until the inversion is rapidly rendered complete.

These processes, in my mind, explain clearly and distinctly all those cases of inversion that appear on record as having occurred after delivery of the placenta, and the patient had been apparently quiet and comfortable from nine hours to two or three days.

They involve essentially three circumstances, namely, a relaxed condition of the womb; an active downward pressure exerted more or less suddenly and forcibly by the abdominal muscles, as in coughing, straining, &c.; and the successive contraction of the circular fibres of the body and neck as the depressed fundus comes in contact with them. Most writers have supposed that in this class of cases, the fundus of the womb was indented during the delivery of the placenta, and the indentation not being immediately removed, the subsequent acts of the patient increased it until it became inversion. But there is no positive proof of such indentation in the history of any of these cases; and we see no necessity of *assuming* its existence at that time, because the same relaxation of the fundus that would allow a simple indentation to remain from one to three days after delivery, would certainly permit any sudden pressure to commence the indentation, de novo, during any part of that time.

We come now to the third and last class of cases of inversion, those that were not known to exist until from nine days to many months after delivery.* To furnish a rational explanation of these cases, it will be necessary, first to state briefly the successive changes that the substance of the womb undergoes from the time of delivery until it resumes its primitive unimpregnated state. We are taught by writers, and the same accords with our own observations, that in ordinary cases the womb continues to diminish by the contraction of its muscular fibres for about four days after delivery. That by such contraction it is diminished from a size sufficient to contain the whole child and surrounding water, to about the size of a child's head with a cavity sufficient to contain only an ordinary hen's egg.

(The witness here drew another diagram upon the blackboard, much smaller than the former ones, to illustrate his idea.)

Suppose the active contraction of the womb has brought it down to something like this in comparison with the others. That is, about the size of a child's head. In the natural process this is as far as the simple muscular contraction is capable of carrying it, and then commences what is supposed to be the process of involution. This process of involution consists in a gradual contraction of the womb, by which, what was previously muscular fibres appear to become somewhat degenerated into a substance resembling fatty matter. As this change of structure goes on, and the proper muscular fibres diminish in number, and gradually disappear, the uterus gradually contracts, and keeps on contracting for a time, varying from about two weeks to that of six or eight weeks. Some authors put it, until it is contracted down to very nearly its original normal size, so that its cavity becomes very small, and its substance com-

*See Cazeaux's Midwifrey, p. 918, case of Ana. and Boudelocque. Also Medico Chirurgical Trans. Vol. 35 p. 145, Cases of H. Davies ; Also, Provincial Med. Journal Vol. 1, case of W. J. Squire ; Also, Dewee's Midwiferey, p. 484, Case of Teallier ; Also, Chicago Medical Journal, Case of A. Fisher ; Also, American Journal of Medical Sciences for Oct. 1860, Case of Dr. Mackey, reported by Dr. J. P. White.

pact and hard as in its normal condition. From a careful study of those cases that appear to have been known, as having occurred only at an advanced period after delivery, it seems to me that there is a process by which they can be explained without impeaching the correctness of the diagnosis or the faithfulness of the description given; take this womb, just as it is contracted there right after the process of delivery. We know that the lower part or mouth of the womb naturally remains soft and flaccid, and relaxed for a longer period than the body. Every accoucheur or obstetrician who has had occasion to examine the fundus of the womb through the abdominal walls, finds it within one or two days to be very firm, so as to feel like a hard ball. If he has placed his finger in the vagina for any purpose, to feel of the mouth of the womb, he finds that at the same time that the fundus is contracted to a hard ball, the mouth is still very flaccid and soft, and easily dilated; from this we infer that the fundus undergoes more rapid condensation than the mouth. But suppose the same process that may cause an atony in the lower part of the womb immediately after delivery, continues to influence the womb in other cases at a later period, and that the lower part which is supplied with nerves from what is called the hypogastric plexus, while the fundus is supplied from another source,—suppose this lower part supplied by that set of nerves, fails in the process of involution. It diminishes as far as muscular contraction will carry it and stops. Suppose that the process of involution either actually never goes on, or goes on very slowly while in the main part of the body and fundus, the involution goes on as usual. The result will be that in the course of a week or ten days, we cannot fix the time, for it may vary a week or two weeks, the involutionary process will have contracted the whole fundus down to its original condition. But if the lower part remains in its uncontracted state, it would bring the fundus, instead of the arched shape, seen in the diagram, almost into a horizontal line, shortening it down, so that it would stand on a line very much less than the lower part of the body and neck. [Witness here drew a diagram to illustrate the disproportion between the contracted fundus and the neck in which the involution had been arrested or retarded.] That of itself would not be sufficient to produce inversion, or even depression of the womb; but when the woman had got to that period, which would be from nine or ten days to three weeks after delivery, according to the rapidity of the change, she would begin to get up, begin to be in an upright position, or should cough much, or it was found necessary to give her physic, and in its operation, should get up and strain at stool, or in passing water—any of these acts with the involutionary process completed or far advanced at the fundus, and arrested at the neck and mouth, would be sufficient with the womb in that condition, to produce a depression and commence an inversion. And that depression once commenced by simply starting here, at the point where the involutionary process was retarded, the whole body would sink down into the vagina, as it were, like the intussusception of the intestines. Intussusception is where any hallow organ, with a muscular coat, a portion of which contracts like a band, and draws it in, so that it is, at a given point, smaller than below, and hence readily become enclosed in the larger part. When it once is drawn in it will go on, until a large part will be invaginated in the part below. Now if the womb here at the junction of the original neck with the body, is contracted more than the part below, that point would be the one where it would begin to depress, and once commenced the forces above, would carry it on more or less gradually, according to the manner in which they were exerted, their frequency in the acts of straining in the evacuation of the bowels, &c., until this upper part would be insinuated into the part below. As it became insinuated into the part below, the vessels would meet a little obstruction, and there would be some slight hemorrhage. The presence of this part crowding on the lower part, would act as a stimulant, and excite more or less contraction in the parts below, which would tend to force on the fundus, when once grasped in them, until it was expelled into the vagina. You might find it merely partial, lying upon the mouth or partially through it, or completely through it, according to the action induced. These are the three methods by which I believe that inversion of the uterus, not only may take place, but the methods by which I believe it has taken place in the numerous cases on record. [The witness here left the black-board, and resumed the usual witness stand.]

*Q.* From the statement you received from Mrs. Stone and her mother, how in your opinion, was that inversion caused?

*Dr. Davis.* Is it allowable for me to state what my opinions were then, and what they were after subsequent investigation?

*Mr. Roberts.* Both, yes sir.

*Dr. Davis.* At the time I was called there, I had not turned my attention to a thorough investigation of the recorded cases, so as to inquire into the symptoms and manner in which they had occurred. And at the time I was there, the only explanation that suggested itself to my mind, was, that there had been a depression of the fundus, at some time, perhaps early after confinement—just a simple indentation—and that it had remained so until her getting up; that when she got up, the weight of the abdominal viscera acting upon it, aided by the acts of singing and so on, that they told me about, had increased the depression, so as to finally produce inversion. But on a close and careful examination of the cases, and more careful reflection upon them, there were certain difficulties in that explanation which made it unsatisfactory to my mind. For instance, the supposition that the fundus of the womb could remain indented for any length of time, commencing soon after delivery when it was large, would pre-suppose that the fundus must also remain relaxed for a length of time, and this could hardly take place without an unusual amount of hemorrhage all the while.

Another difficulty in the way was, that in this particular case, as well as in all the parallel cases, the womb itself, as I found it, was hard, and the fundus was firm, as though it had been contracted down to its natural size, and this was inconsistent with the idea that it had been relaxed all the while, as it would be in a case of a dimpling that would continue for that length of time; consequently I began to compare this case with other cases, such as reported by W. J. Squires, in the

Provincial Medical Journal, and others. I also studied more closely the particular phenomena or symptoms that occurred in the several cases. This investigation satisfied my mind, that instead of these late cases, the inversions that are produced by, or start with a simple depression of the fundus immediately after delivery, are those in which it becomes complete, either within a few hours, or a couple of days after the delivery; that the indentation must go on to inversion or in the process of the natural contraction of the womb, the dimpling must rectify itself spontaneously ; and that the case of Mrs. Stone, belonged to the same class with the one by Squire. The woman had not had any trouble ; the afterbirth came away spontaneously ; no hemorrhage ; and she remained entirely comfortable until the night of the 8th day, when she dressed herself and got up. That case is reported by Lee ; it was originally reported in the Medical and Surgical Journal. After she had got up, having dressed herself, she being then comfortable, there was a slight flowing. Two days afterwards, when she was up, a more decided flowing of blood took place, estimated as the reporter states, to be near a quart of blood. From that time she continued to suffer from flowing, not all the while blood, but either a bloody fluid or a discharge of a mattery substance, and mucous from the vagina. She continued weak, and grew more and more exhausted until the 21st day, when the examination was made, and the womb found completely inverted. This is only a sample of that class of cases. Another is reported by Ane, in which the woman, after going on comfortably as far as anything can be learned from the report, until the 12th day, when she got up, and while straining at stool to get an evacuation of the bowels, symptoms more or less marked, occurred, which soon led to an examination of the womb, when it was found to be inverted. There are four or five other cases of similar character, which had led me to suppose, from the fact that the flowing had ceased, that there must of necessity have been contraction of the uterus. There was no flowing, and no vaginal discharge to indicate excessive irritation in Squire's case, until after the tenth day. It seems to me, there can be no explanation given that will account for the symptoms in that case, except that the process of involution had gone on unequally, the woman feeling no inconvenience from it until she assumed the upright position, and there came to be pressure upon the parts, when the first step towards inversion took place with a slight flow; still not enough to create alarm, until she is up two days afterwards, when a more copious flow took place ; then she began to suffer from pressure below; constant flowing with continued weakness and retention, yet not so marked and decided an obstruction of the passages of urine, or the fœces, as to cause alarm. She goes on with these vaginal discharges until the 21st day, when the uterus was found completely inverted. After a close and careful comparison of the cases, I become satisfied that in Mrs. Stone's case, instead of its taking place by indentation at the time of delivery, all the symptoms that they detailed to me, indicated that the womb had contracted, that it had been felt above the pubes, and that finally the hemorrhage had ceased, that she had began to set up, and that the return of flowing came on gradual day by day, and little by little. From all this, I became satisfied that it belonged to this third class of cases where the womb is inverted by inequality in the involutionary process, by which the fundus of the womb had become shortened and contracted more in proportion than the neck; and not only shortened, but rendered more horizontal instead of arched, thereby allowing the exterior pressure to commence a depression. I must say that after very thoroughly and carefully studying those cases of Ane and Bodeloc-que and Squire, I cannot see any way to account for a mistake in diagnosis. I credit them, and I believe they are true. In Ane's case, it is reported that he had examined the womb carefully. My reason for crediting these cases, is, that they are reported by men whose veracity has never been questioned, and whose skill is equal to any in the profession, and if we discredit them, we must discredit the whole profession. I remember Desormeroux, case 21st reported in Cross. Desormeroux was a man of considerable eminence as a physician and accoucheur. I remember, and made a minute of Forbes' case, reported in Braithwaite, which was first detected four days after delivery; also, Teiller's case, reported in Braithwaite, ten days after delivery, copied by Dewees. I credit that case because there is nothing in the narrative, or connected with it, to lead me to doubt it. I remember Forbes' case, 27 and 28 Braithwaite, p. 266, 1850. I think it is the same case which was reported in the Medico-Chirurgical Transactions, and copied by several others. In that case the reporter states that there was no difficulty in removing the placenta; no untoward symptoms; and specifically that after the delivery of the child, and the placenta, both, for three days the uterus was well contracted. I have read Teals' case, but have made no memorandum of it, because that and several others were discovered at remote periods, and no facts to identify at what time they occurred. I cannot remember the names of all these others.

*Mr. Van Arman* objected to the cases being given in detail by the witness.

After argument by counsel, the court decided that the question might be asked whether inversion might occur under certain circumstances. If yea, what was his reasoning for it; and that reason becomes evidence before the jury, but if he says so because it is reported, it will be of little weight.

*Q.* Do you think it possible or probable that a womb could become inverted nine days after an abortion at the fourth month.

Objected to as irrelevant. Objection overruled; defendant's counsel excepted.

*A.* Under the third explanation of inversion that I have given, I think it would be possible. Such a thing would be much less probable than at later periods. The womb at the fourth month usually varies much in different individuals. I have already answered as to whether I think inversion probable nine days after abortion at the fourth month; I think it possible; I think it has occurred; my opinion is the inversion in Mrs. Stone's case commenced when Mrs. Stone began to get up; about the time that she began to set up in the chair without being lifted up out of bed; about the time she began to set up sufficiently strong to be wheeled across the room. I think that was about the end of the third week, or the commencement of the fourth. From the absence of sudden and severe symptoms and the gradual continuance of the flowing, and the consequence connected with it, my opinion is, that the inversion went on gradually. As near as I can fix upon the facts which would sustain or point to any period of time when inversion became complete, it was at the time or immediately after she was up the last time singing loudly, when I think they stated, she had a more decided flowing. I think the inversion became complete after she was at the piano singing loud and clear. If it had been going on gradually, the symptoms would then have been simply sensations of faintness, weakness or prostration, with a greater amount of hemorrhage than had preceded it during the earlier stages of the inversion. The symptoms are not in all cases uniform, but these would be most likely.

Mr. Roberts proposed to ask the witness whether he had read certain cases, and whether he credited the same. Defendant objected. Objection overruled. Defendant's counsel excepted.

*Q.* Have you ever read Skae's case, reported in Crosse, and if so, do you credit it?

*A.* I have read it, and credit it, certainly.

*Court.* Do you believe it might take place, and become complete at a period 2½ years remote from parturition?

*A.* I think it is possible; on the third theory that I suppose, we cannot make any limit to the time. It is a question of the condition of the organ, not a question of time.

At the time of delivery, the womb is large, and it is very vascular. The vessels that exist between it and the afterbirth, are also large, and an actual inversion in that condition, if the afterbirth is detached, or partially detached in the act, almost necessarily involves either very great and alarming flooding and loss of blood, with rapid signs of sinking and syncope, or there may be only a rush of blood, and the syncope becomes so quick and so profound, that the blood is stagnated in the vessels, and the flooding prevented; or if the afterbirth continues attached, there may not be flooding, but the shock that is produced, will be very great. From the size of the womb, the change which it necessarily undergoes, must produce a very alarming condition of the patient—very strong symptoms, such as extreme faintness, great sense of distress, or severe flooding or both.

Mr. Roberts, here put the Hypothetical case, that in addition to the facts early testified to by the witness, "that about the time the afterbirth came away, the patient uttered an outcry as though in great pain, such as, Oh, Dr. what are you doing to me," &c.? Flowing more than ordinary, but not enough to produce syncope, &c., &c. As put to other witnesses, giving symptoms up to 10 o'clock on the night of delivery, would your opinion then be modified, as to whether inversion took place at the time or not?

*A.* No Sir, it would not, because the fact that the afterbirth was delivered, shows that the vessels were actually ruptured, and the womb being then inverted, and remaining in that condition without being immediately replaced, would have produced inevitable flooding of a very alarming character, or syncope and exhaustion. If it had been immediately replaced, the afterbirth being thrown off, it might possibly have been accomplished, so as to have avoided sufficient hemorrhage to have induced syncope, but then the shock would have been very great. The exclamation you speak about, Oh Dr. you hurt me, is a very common one with women in their first labor; so far as my experience goes with two thirds of all the women I put to bed, especially with the first child, and sometimes with the subsequent one, when the cord is tied and the child carried away, and the finger carried back into the vagina to ascertain where the placenta is, they shrink and say, Dr. what are you doing, or Dr. you hurt me. Such statements are so common at the time we are feeling for the afterbirth, or ascertaining where it is that we regard them of no account. If the uterus were dragged down with the afterbirth still attached, the shock to the system would be such as to produce prostration, coldness of the extremities, failure of the pulse, and all the symptoms of rapid and speedy dissolution, from which the patient might die in an hour, or might recover, but the shock would be very severe. If the womb was inverted as the afterbirth was detached, it would pour out an amount of blood, that would be excessive and alarming, and would continue to do so until the patient was exhausted, or so perfect a syncope took place as to arrest the blood in the vessels and allow the coagula to form, from which a patient might recover and might not.

As the womb ordinarily contracts in natural labor, it is found that a force sufficient to tear the cord off, will not invert it. I have sometimes made pretty firm traction on the cord, when the womb was contracting well, and seemed in good condition. I have had some cases where the afterbirth was pretty strongly adherent, and rather than introduce my hand into the womb, and finding that the walls were firm, I have made as strong traction as I could without tearing it, and then carried the hand into the womb, and peeled it off with the fingers. I believe the womb to be susceptible of inversion by traction on the cord, must be in a state of entire atony in the lower half of it, or probably the whole of it; but if it should happen that the fundus alone was contracted, at the same time that the lower part was inactive, then the pulling and the contraction would coincide to start the inversion. I can conceive of sufficient force being applied in that way, with the womb in an entirely passive condition to invert it, but not when it is in an actively contracted condition. The way I understand the rule, which I believe is a correct one, is that no traction, except it be very slight, should be made, unless the attending physician is at the same time certain that the womb is in a state of tension, or the walls firm; that is the common practice, I think. I do not know how others will take it, but certainly I understand it to be the common practice, that soon after the child is laid aside, and the cord is severed, the accoucheur, or attending physician takes hold of the cord with the fingers of one hand, and either passes the other up under the clothes over the pubes, so as to ascertain the condition of the fundus of the womb, or he carries the finger of this other hand along the cord up into the vagina, to ascertain whether the same pain that expelled the child, has not detached the afterbirth, and left it lying in the mouth of the womb, or partly in the mouth, and partly in the vagina, or wholly in the vagina. Either or both these modes are practiced by the same physician. If in passing the finger up into the vagina, and tracing the cord, it is found that the afterbirth is not in the vagina, nor engaged in the mouth of the womb, and there is no flowing to show that it has been detached, the accoucheur acts on the presumption and certainty that it is still undetached. In that state he would make no traction on the cord, except just to hold it in his fingers as a guide to know whether it was moved by the action of the uterus, or not; but he would withdraw the other hand from the vagina, and carry it over the pubes, and make a little rubbing or friction to excite the womb to contraction, or if he had an intelligent nurse by, he might keep his finger in the vagina, watching the progress of things there, and ask the nurse to make a little friction. Sometimes the patient herself is asked to make a little friction there to excite contraction. These things usually bring about a contraction, which we call a pain; the afterbirth is detached, and as quick as it is, out comes a gush of blood; that gush of blood warns the practitioner that the afterbirth is detached. The fingers that hold the cord, find, in just keeping it straight, that the same act that caused the gush of blood, has left the cord slack, and he then makes a slight traction, and if it is already loose, it comes along; or it may be that the force of the pain is sufficient to expel it without traction, and he finds it coming down upon his hand and finger in the vulva, and it comes away without straightening the cord. But more generally the contraction of the womb expels it into the vagina, leaving parts of it still enclosed in the mouth of the womb, and it requires a little traction on the cord or hooking the finger over the edge of it, to bring it away; either of these acts will frequently cause the patient, the vagina being sensative from previous distension and labor, to complain, "you are hurting me." But the practitioner should not make any traction on the cord, more than barely to straighten it. No force should be applied to it, unless he is confident that the womb is in a state of tension. He should not make traction, when it is in a state of atony. I

have already stated what the symptoms would be of inversion, either by traction on the cord, or by the hand of the accoucheur manipulating for the placenta. I now include manipulating with the hand. If he was manipulating the placenta, it must be with the hand in the womb, if it were still attached, because manipulating in the vagina, could not act upon the placenta. If the placenta is down where you can manipulate upon it in the vagina, it is then already detached, or there is an inversion already existing. But if his hand grasps it in the womb, instead of insinuating his fingers so as to separate it, he might grasp it between his thumb and fingers, and merely pull, and readily pull down the fundus and produce inversion. The symptoms of that would be the same as would occur in any other way; it would be a very severe shock to the patient; inducing sinking, syncope, exhaustion, and signs of dissolution; or if the afterbirth were afterwards finally detached in the act, he would find after this, rapid flowing, until a degree of faintness would arrest it. I think the ordinary attendants would be aware of it, as it would inevitably attract attention, and create the most intense alarm, unless the practitioner was so skillful that he could put it back before they had found out that anything serious had happened. I think a nurse 30 years of age, a married woman who had borne children, but not living children, having about eight year's experience in nursing, would be able to feel the womb above the pubes readily. She might not be competent to decide positively, but she would be able to feel the tumor there. She might not be able to decide whether the tumor she felt was a womb or a distended bladder, both occupying the same region. She might have considerable experience as a nurse, and not know the one from the other, unless she knew that the patient had evacuated water regularly, then she would know it was not the bladder. Almost every nurse of ordinary intelligence, knows where the womb is after delivery; they cant help it, in adjusting the bandage they come in contact with it, and in changing the clothes. They detect its existence until it is so far contracted as to sink below the level of the pubes, which is usually after the fourth day, sometimes varying; it may not entirely disappear from above the pubis for eight or ten days, but that would be very slow. Women who have their first child, *prima paries*, find a lump there, and they dont know what it is; a good many times they have asked me what it was; they have had no experience about it. If inversion occurred along about the third week, I think the process of involution at the fundus would have contracted it to the size that I found this one, and consequently on account of that contraction, would have limited very much the amount of hemorrhage that would have taken place in connection with the inversion; the vessels would have been reduced so small in the fundus, that they would bleed much less freely than at an earlier stage. This involutionary process is sometimes retarded.* It is very variable in the rate of its progress, but the exact causes which produce that variation, we are not able to define. We suppose a lax temperament or weak organic movements, where anything had previously depleted the patient, might be a cause. But the causes are not clearly definable, any more than you can tell why one man, accustomed to a particular mode of exercise will develope his muscles faster than another who seems to follow the same mode; there are different modes with different individuals about their organic processes, that makes them take place in a different ratio, that we cannot explain. The reasons why the symptoms, at the time that this inversion took place, as I believe, did not indicate it more violently, have been already given in the fact that on the theory I have supposed, the fundus had gone on in the process of involution, and consequent diminution of size, so that its vessels were already small, and there was nothing to occasion the rapid pouring out of excessive quantities of blood, so as to induce faintness or exhaustion, as at an earlier period. If it went gradually on, as different successive acts caused pressure to be made upon it, the shock at no time would be great, the smallness of the organ would lessen the severity of the shock, as well as diminish the amount of flowing. If we suppose that the process of involution going on

---

* Boivin and Duges, say the process of involution may be retarded from TWO to SIX MONTHS.

unequally as I have mentioned, the dimensions of the cavity of the organ, longitudinally would be reduced to the natural one, that is, two or two and one-fourth inches; while the transverse dimension of the cavity, by a failure of the involutionary process in the lower parts, would be considerably larger than that. It might be an inch, or even vary from one to two inches in diameter. It would depend entirely upon the extent to which the involutionary process had been retarded, or the period of time when it was entirely arrested. The circumstance of her actually fainting on the fourth day, when this sinking spell occurred, would not alone alter my opinion, nor the circumstance that they used volatile salts in restoring her; that has no meaning at all. Salts are used for headache, and almost everything. Bottles of hot water to the side and feet would have no influence to make me think there was inversion at that time, because they are simply indication that the patient has coldness at the extremities, or something approaching chilliness. I would want a much more rapid flowing, a severe flowing, as the placenta had already been removed, to think that the womb had been inverted at that time, on the fourth day. I should expect a loss of considerable blood accompanied by syncope, and the womb would have been large enough to have filled the whole of the vagina, if it had not protruded, the womb being on the fourth day as large as a child's head. Independant of difficulty in the acts of urination and defecation, the patient would be inclined to strain, and these acts would have demanded interference to relieve the patient in a few hours. If it lasted but two hours, and she recovered, I think she would have complained of the bearing down pains. I think the womb of the size of the fourth day, could not be contained in the vagina, without producing bad feelings, which the patient would complain of directly. If the womb was inverted by traction on the cord, in removing the afterbirth, and remained inverted so soon after removing the afterbirth, which is usually from ten minutes to an hour after expulsion of the child, it would be much more enlarged than in cases where everything has gone on well; and I don't think a womb in a state of relaxation, within the first two hours after the expulsion of the child, especially with the afterbirth added to it, could be contained in the vagina. I think if it were a complete inversion, it would necessarily protrude, because the dimensions of the organ itself, at that period of time, with the placenta added, are much greater than the ordinary dimensions of the vagina. If the physician had detached the afterbirth and pushed it back into the vagina, it would have produced flowing, and obstructed the passage, and in addition to that, there would be fœtid sanguineous, mattery discharges ; a kind of mixture of bad smelling mucous with blood. That would occur if it were left there two or three days, but not at first. If the attendants did not know it was there, they would know that something was the matter with the patient ; that she could not make her water, or that this discharge which was very unusual, was going on. They might not, if it were placed back in the vagina, and sufficiently squeezed to retain it there, positively know that there was an inversion, because the attendants are not supposed to make any vaginal examination, but they would know there were impediments to these ordinary functions, and that the woman continued to have unusual discharges. The woman would not in my opinion be able to be playing on the piano and singing on the 21st day, because I think the inversion continuing to that time, would have exhausted her to such a degree, by the hemorrhages and discharges, that the moment she attempted to get up, the sense of dragging would have been so great that she would have had no ability or disposition to sing. It would not necessarily destroy the appetite, but it would perhaps induce weakness that would impair it. My opinion would not be modified in any way from what I have expressed it, if the nurse had felt the womb at the hypogastrium on the fourth day, only, if I were satisfied that she found it there, it would add to the evidence in my mind, that it was not inverted at the time of delivery or near that.

Court adjourned.

THIRTEENTH DAY, THURSDAY, NOV. 8th, 1860.

Dr. N. S. Davis recalled and further examined by Mr. Roberts.

*Q.* From the history of Mrs. Stone's case, as you derived it from herself and her mother, state whether in your opinion, negligence or want of skill on the part of the physician in attendance, contributed to the inversion, or whether he was negligent or unskillful, in not discovering it sooner?

*A.* I did not learn any facts that indicated neglect or want of skill, in the progress of the case.

Mr. Van Arman objected to an examination of the witness, except solely as an expert.

*Q.* What are the symptoms of indentation?

*A.* The symptoms that have been found to accompany indentation, I think vary very much. It is sometimes accompanied by an excess of flowing, and at other times by very slight symptoms of any kind. In partial inversion, I mean going forward from indentation in the stage between an indentation and that of complete inversion, there is usually some degree of flowing which varies very much in its amount, feelings of fullness, and usually some bearing down and straining, a dragging sensation. In reference to Leverett's case and others, years after parturation, I can only express the opinion, that there is nothing in the history that is given, showing that they manifested active symptoms. I have met with cases of hemorrhage that I supposed would be classed as secondary hemorrhage, at all periods, from two weeks to three months after delivery. Secondary hemorrhage is supposed to be occasioned in many instances by simple relaxation, want of tone in the tissues of the womb. In Mrs. Stone's case, from the facts that they stated to me, I supposed that there was a degree of laxity of the fibres of the womb, that predisposed to flowing. I inferred that from their statement to me, that she had had an abortion previously, and partial prolapsus, and supposed that the uterus was in an irritable condition or predisposed to hemorrhage. I placed some importance on that abortion, from the fact that women who have been known to abort or miscarry, so far as my observation goes, are invariably more disposed to hemorrhage and vaginal discharges, at any subsequent period after delivery, than those who had not. The question how long a physician should wait before making a vaginal examination in a secondary hemorrhage after delivery, depends in my opinion entirely upon two questions. That is, the profuseness or rapidity of the hemorrhage on the one hand, and on the other, whether it is accompanied by symptoms such as bearing down, fullness low down, pressure upon the urethra, or obstructions to the discharges. A simple moderate hemorrhage alone without other symptoms, I should infer, depended upon general causes, or simple relaxation, and confess that I should not be disposed to insist upon an examination without using the ordinary remedies for a length of time that would depend entirely upon their effect. If in using them, the hemorrhage seemed to cease, or nearly so, I should expect it was going to cease entirely, that is, upon the supposition that there were no other marked symptoms. If I did not make any impression upon it, and it continued steadily, I should be disposed to insist upon an early examination. Consequently, it is totally impossible to fix upon a period of time that the practitioner would be justified in waiting. He would be obliged to be guided by his own judgment and the accompanying circumstances.

